# Chemotherapy treatment patterns, costs, and outcomes of patients with gastric cancer in the United States: a retrospective analysis of electronic medical record (EMR) and administrative claims data

**DOI:** 10.1007/s10120-015-0486-z

**Published:** 2015-03-20

**Authors:** Lisa M. Hess, Diane Michael, Daniel S. Mytelka, Julie Beyrer, Astra M. Liepa, Steven Nicol

**Affiliations:** Eli Lilly and Company, Indianapolis, IN 46285 USA

**Keywords:** Stomach neoplasms, Outcome assessment, Economics, medical, Retrospective studies

## Abstract

**Background:**

The aim of this study was to conduct a retrospective database analysis to describe the chemotherapy treatment patterns and outcomes of patients with gastric cancer.

**Methods:**

Individuals diagnosed with gastric cancer were identified from the IMS Oncology Database, which contains electronic medical record (EMR) data collected from a variety of community practices, and the Truven Health MarketScan^®^ Research database, an administrative claims database. Eligible patients were 18 years of age or older and had an ICD-9 code 151.0–151.9. Patients were excluded if they had evidence of cancer within 6 months of the index diagnosis.

**Results:**

There were 5257 eligible patients identified in EMR data: 1982 (37.7 %) of these patients also had data regarding chemotherapy treatments. Of the 1982 patients who received first-line therapy, 42.3 %, 18.1 %, and 7.9 % went on to receive a second, third, and fourth line of chemotherapy, respectively. There were 11891 eligible patients identified in the administrative database; 5299 (44.6 %) had data regarding chemotherapy. Of those initiating chemotherapy, 2888 (54.5 %) received a second line and 1598 (30.2 %) received a third line of treatment. The average total cost of care during first-line therapy was $40,811 [standard deviation (SD) = $49,916], which was incurred over an average of 53.5 (SD = 63.4) days. A similar pattern was evident in second-line treatment (mean/SD, $26,588/$33,301) over 41.2 (SD = 55.7) days.

**Conclusions:**

Costs and duration of care received vary among gastric cancer patients in the U.S. There is a need to understand which regimens may be associated with better health outcomes and to standardize treatment as appropriate.

## Introduction

Gastric cancer is the 5th most common cancer worldwide, but is relatively less common in the United States (U.S.), where it has the 16th highest incidence rate of all cancers. In 2014, it is estimated that 22,220 new cases of gastric cancer were diagnosed and 10,990 patients died of gastric cancer [1]. Although those diagnosed with early-stage disease may be cured of their disease, the prognosis for most patients is poor. The 5-year relative survival rate for patients diagnosed with localized disease is 64.1 %, but this rate declines to only 4.2 % for those diagnosed with metastatic disease [2]. Unfortunately, 80–90 % of patients are diagnosed with advanced-stage disease [2] when surgery and local therapies are no longer effective.

For patients with advanced or metastatic disease or for postoperative therapy, the NCCN (National Comprehensive Cancer Network) guidelines currently recommend the use of platinum plus fluoropyrimidine as first-line therapy [3]. Despite treatment, many patients experience disease progression or recurrence. After progression or recurrence, limited therapeutic options were available until 2014, when the NCCN guidelines were updated to include the preferred use of single-agent ramucirumab (Category 1 evidence) with the existing recommendations for single-agent chemotherapy (e.g., paclitaxel, docetaxel, irinotecan) [3]. Although data are not yet available related to the real-world use of ramucirumab, the data from claims and electronic medical records can inform practitioners and researchers regarding the care and cost of individuals diagnosed with gastric cancer.

The primary objective of this descriptive study was to explore chemotherapy treatment patterns, healthcare resource utilization, costs, and outcomes for patients in the U.S. diagnosed with gastric cancer in an electronic medical record and administrative database, respectively.

## Methods

### Data sources

Electronic medical record (EMR) data were obtained from the IMS Health Oncology Database, which is an integrated database consisting of oncology EMR. The database contains de-identified biomedical data from more than 740,000 cancer patients who received care from approximately 550 providers in 737 facilities, representing cases from all 50 U.S. states.

Administrative claims data were obtained from the Truven Health MarketScan Research Databases, which include person-specific clinical utilization, expenditures, and enrollment across inpatient, outpatient, prescription drug, and carve-out services. The database links paid claims and encounter data to patient information across sites and types of providers and over time, and includes private-sector health data from approximately 100 payers and more than 98 million patients.

Both databases provide longitudinal data from clinical practices as part of routine clinical care across the U.S.

### Eligibility criteria

Patients age 18 or older with a new diagnosis of gastric cancer (ICD-9-CM 151.0–151.9) between January 1, 2004 and March 31, 2012 (administrative database) or between January 1, 2004 and January 1, 2012 (EMR database) were eligible for inclusion. The first occurrence of the eligible ICD-9 code was defined as the “index diagnosis.” Patients were ineligible if they had any evidence of cancer within 6 months before the index diagnosis or if they had any evidence of gastrointestinal stromal tumor (ICD-9-CM 238.1) at any time. Continuous medical benefits for 6 months before the index diagnosis were required for eligibility of patients in the administrative dataset.

### Demographic and clinical variables

Demographic data in both databases include age, gender, diagnoses (ICD-9 codes), and dates of service associated with each diagnosis. The EMR database further contains patient ethnicity, tumor stage, ECOG performance status data, and laboratory tests. The databases also include information on insurance status (EMR data) or insurance type and plan information (administrative data).

### Resource use and cost variables

Administrative claims data include detailed records for hospital inpatient admissions, outpatient medical claims, professional claims (private physician offices or stand-alone infusion centers), and service and facility files, with additional information such as the length of stay at specialized nursing facilities, date and duration of service (e.g., length of stay for hospital admissions) for medical claims, provider type and place of service, and plan payment and patient copayment amounts. The administrative data also include pharmacy claims, which report national drug codes (NDC), therapeutic class of the agent administered or provided, dispense date, the quantity and days supplied, and plan payment and patient copayment amounts. ICD-9, Healthcare Common Procedure Coding System (HCPCS), NDC, and Current Procedural Terminology (CPT) codes were used to identify chemotherapy, radiation therapy, surgical procedures, and other supportive care medications used. Costs were obtained from third-party payment data fields and adjusted for inflation and reported in 2012 U.S. dollars, using the medical care services component of the Consumer Price Index. Because of the possible underestimation of costs and/or resource use data, cost analyses excluded cases with Medicare supplement and capitated claims and resource use excluded cases with Medicare supplement claims. Resource use in the EMR data was limited to therapeutic regimens including each molecule and generic drug name, dose(s), date(s) of administration, and length of therapy. Surgical procedures were identified by HCPCS and ICD-9 procedure codes specific to gastric/gastroesophageal-related procedures.

To simplify the data, individual chemotherapy and biologic agents were collapsed into drug classes as follows: biologic/targeted agents included bevacizumab, cetuximab, crizotinib, everolimus, dasatinib, imatinib, gefitinib, sorafenib, sunitinib, pazopanib, regorafanib, panitumumab, and rituximab. Platinum agents included cisplatin, oxaliplatin, and carboplatin. Taxanes included docetaxel, paclitaxel, and nab-paclitaxel. Anthracyclines included epirubicin, daunorubicin, doxorubicin, and mitoxantrone. Alkalating agents included cyclophosphamide, ifosfamide, melphalan, chlorambucin, mitomycin, and thiotepa. Antimetabolites included gemcitabine, hydroxyurea, methotrexate, fludarabine, cladribine, and pemetrexed. Topoisomerase inhibitors included irinotecan, etoposide, damptothecin, and topotecan. Vinca alkaloids included vincristine, vinblastine, and vinorelbine. Fluororpyrimidines included fluorouracil and capecitabine. Other smaller groupings included folic acid analogues (leucovorin) and hydrazine/triazines (dacarbazine, procarbazine). All other antineoplastic agents were put into the category of ‘other.’ Concomitant medications were similarly grouped into the following categories: hematopoietic agents, transfusions, antibiotics, antivirals, anti-emetics, antifungal agents, anti-infectives, pain medications, nutritional supplements, bisphosphonates, and hormonal agents (complete medication lists available by request from the authors).

### Analysis plan

Patient demographic and clinical characteristics assessed at the gastric cancer index date, depending on the database, included age, sex, stage, race, primary location of tumor, performance status, length of follow-up, and insurance plan status. All analyses were descriptive and exploratory in nature and were conducted using SAS 9.2. All variables were summarized descriptively through the tabular and graphical display of mean values, medians, ranges, and standard deviations (SD) of continuous variables of interest and frequency distributions for categorical variables. No tests of statistical significance were planned or performed as part of this study because it was descriptive in design.

Survival was estimated in the IMS database using the last occurrence of a record in the database as a proxy for date of death of each patient. This strategy has been used previously with IMS data. Patients who reached the end of the database were censored from the analysis. Survival estimates were reported as number of days from the date of diagnosis to the proxy date of death and were reported as mean, SD, range, median, and interquartile range values for the entire gastric cancer population, by stage of disease, and for patients who received chemotherapy. Survival was also estimated for patients who had a second line of chemotherapy from the date of start of the second line of therapy to the proxy date of death.

Average monthly costs were defined as the total per-patient costs divided by number of months within the time period for which the patient’s total costs were calculated. This approach was used because of the varying survival of patients with gastric cancer and different periods of chemotherapy treatment (resulting in inconsistent follow-up periods). Average total cost of care was defined as the all-cause cost for healthcare from the time of the index diagnosis to the end of the database, regardless of the duration of follow-up and regardless of disease status. To standardize differential follow-up time periods, average monthly costs were also reported for the total cost of the care period.

Missing data were included as a categorical field (missing or unknown). No imputation was made to account for incomplete data. Patients with partial data were included in analyses for which there are complete data to retain sample size; as a result, cases were not fully excluded as a result of missing data.

## Results

There were 5257 and 11,891 patients identified meeting all eligibility criteria in the EMR and administrative claims datasets, respectively. Table 1 presents the baseline demographic and clinical characteristics of the two sets of patient data. This table demonstrates that there were considerable missing data in the EMR system for disease stage, performance status, and insurance status. Additionally, the two data sources collect slightly different types of information (e.g., clinical information is recorded in the EMR data whereas resource utilization and cost data are only available in the administrative data).

**Table 1:** Patients eligible for inclusion

Characteristic	Electronic medical record (EMR) data	Administrative claims data
	*N* = 5257	*N* = 11,891
Mean age (SD)	64 (13)	65 (13.9)
Gender, *n* (%)		
Male	3197 (60.8)	7427 (62.5)
Female	2059 (39.2)	4464 (37.5)
Unknown	1 (0.02)	0 (0)
Health plan, *n* (%)		
Commercial	59 (1.1)	5881 (49.5)
Medicaid	104 (2.0)	–
Medicare	4 (0.1)	6010 (50.5)
Unknown	5090 (96.8)	0 (0.0)
Specific plan type, *n* (%)		
Comprehensive	–	3217 (27.1)
Exclusive provider organization (EPO)	–	90 (0.8)
Health maintenance organization (HMO)	–	1773 (14.9)
Non-capitated point of service (POS)	–	694 (5.8)
Capitated point of service (cPOS)	–	73 (0.6)
Preferred provider organization (PPO)	–	5373 (45.2)
Consumer-driven health plan	–	163 (1.4)
Unknown		508 (4.3)
Site of gastric diagnosis, *n* (%)		
Malignant neoplasm of the stomach (ICD-9, 151.x)	1 (0.02)	243 (2.0)
Malignant neoplasm of the cardia, including cardiac orifice, cardio-esophageal junction (ICD-9, 151.0)	751 (14.3)	3695 (31.1)
Malignant neoplasm of pylorus, including prepylorus, pyloric canal (ICD-9, 151.1)	56 (1.1)	0 (0.0)
Malignant neoplasm pyloric antrum, includes antrum of stomach NOS (ICD-9, 151.2)	277 (5.3)	987 (8.3)
Malignant neoplasm, fundus of stomach (ICD-9, 151.3)	215 (4.1)	346 (2.9)
Malignant neoplasm, body of stomach (ICD-9, 151.4)	567 (10.8)	970 (8.2)
Malignant neoplasm, lesser curvature, unspecified (ICD-9, 151.5)	190 (3.6)	238 (2.0)
Malignant neoplasm, greater curvature, unspecified (ICD-9, 151.6)	116 (2.2)	189 (1.6)
Stomach, unspecified, including carcinoma ventribuli, gastric cancer (ICD-9 151.9)	2689 (51.2)	4506 (37.9)
Stage at diagnosis, *n* (%)		
Stage 0	7 (0.1)	–
Stage I	251 (4.8)	
Stage II	295 (5.6)	–
Stage III	345 (6.6)	–
Stage IV	682 (13.0)	–
Unknown	3677 (69.9)	–
Charlston Comorbidity Index score at index diagnosis, mean (SD)	–	2.58 (2.81)
ECOG performance status, *n* (%)		
0	331 (6.3)	–
1	444 (8.4)	–
2	156 (3.0)	–
3	29 (0.6)	–
4	2 (0.04)	–
Unknown	4295 (81.7)	–
Duration of follow up from index diagnosis, mean (SD) days	607.4 (652.6)	577 (607)
Evidence of chemotherapy, *n* (%)	1982 (37.7)	5299 (44.6)
Lines of therapy (of those receiving chemotherapy)		
Mean (SD)	1.76 (1.28)	–
Received 1 or more lines, *n* (%)	1982 (100)	5299 (100)
Received 2 or more lines, *n* (%)	838/1982 (42.3)	2888/5299 (54.5)
Received 3 or more lines, *n* (%)	358/1982 (18.1)	1598/5299 (30.2)

*SD* standard deviation, *ECOG* Eastern Cooperative Oncology Group, *NOS* not otherwise specified

**Fig. 1:**
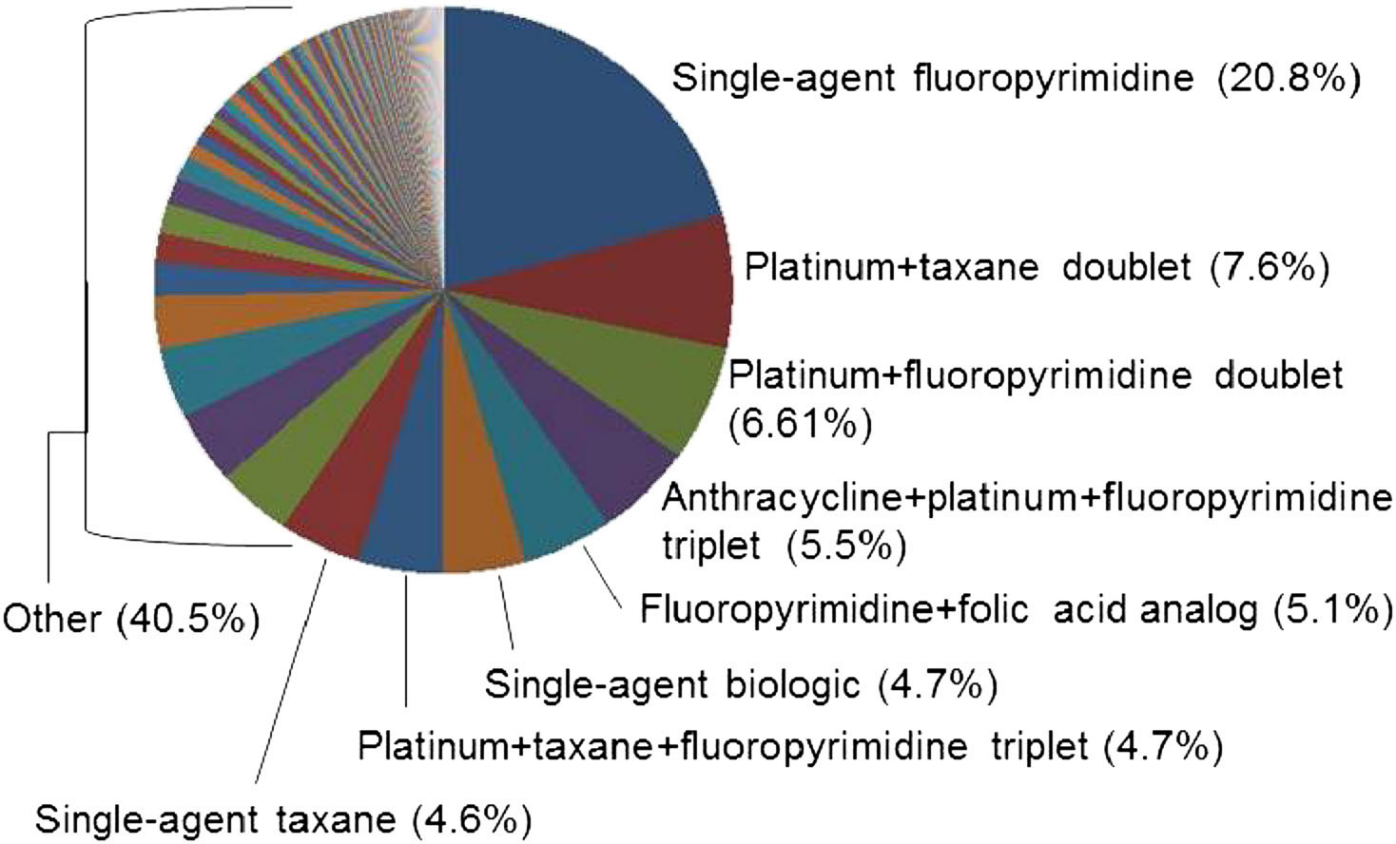
Regimens used in second-line gastric cancer: administrative claims data (*N* = 2831)

There was evidence of chemotherapy treatment for 37.7 % (*n* = 1982) and 44.6 % (*n* = 5299) of patients in the EMR and administrative data, respectively. In both databases, most patients were treated with platinum- and/or fluoropyrimidine-containing regimens in the first line (89.7 % and 88.7 % in the EMR and administrative databases, respectively). Of patients receiving first-line chemotherapy, 42.3 % received additional lines of therapy in the EMR database, and 54.5 % received additional lines of therapy in the administrative database. In the EMR database, of all patients who received first-line therapy, 838 (42.3 %) went on to second-line treatment, 358 (18.1 %) went on to receive third-line treatment, and 157 (7.9 %) went on to receive a fourth line of therapy. In the EMR database, a total of 131 unique drug combinations were identified in second-line therapy, and in the administrative data, 351 unique drug combinations were used in the second-line setting. The unique combinations were collapsed into drug class groupings and are summarized in Fig. 1 and in Table 2, which demonstrate the consistency in the classes of drugs used by line of therapy for gastric cancer between the two data sources, despite the variety in drug combinations used in the second line. There was no standard treatment regimen that was commonly used following the first line of therapy; however, the same agents used in the first line tended to be used in the second line in a wider variety of combinations. Although irinotecan-based treatment occurred in 284 (9.8 %) as second line, it was used inconsistently with a wide variety of combinations (e.g., the most common combinations included platinum, fluoropyrimidines, and/or taxanes). The wide variation in irinotecan use resulted in the finding that no specific irinotecan-based regimen was used in more than 2.5 % of the study population (these regimens are all in the ‘other’ category of Fig. 1). Post hoc analyses exploring trends in treatment patterns could not identify any clear changes from the earlier time period (2004–2009) to treatment in 2010 and later; treatment patterns remained heterogeneous throughout the study period.

**Table 2:** General classes of chemotherapy agents (number and percent) used alone or in combination

	Electronic medical record (EMR) data	Administrative claims data
**First-line therapy,** ***n***	**1982**	**5299**
Biologic, *n* (%)^a^	154 (7.8)	435 (8.2)
Taxane, *n* (%)^a^	495 (25.0)	1505 (28.4)
Anthracycline, *n* (%)^a^	408 (20.6)	880 (16.6)
Fluoropyrimidine, *n* (%)^a^	1499 (75.6)	3521 (66.4)
Platinum, *n* (%)^a^	1095 (55.2)	3294 (62.2)
**Second-line therapy,** ***n***	**838** ^c^	**2831** ^c^
Biologic, *n* (%)^b^	103 (12.2)	340 (12.0)
Taxane, *n* (%)^b^	243 (29.0)	742 (26.2)
Anthracycline, *n* (%)^b^	137 (16.3)	381 (13.5)
Fluoropyrimidine, *n* (%)^b^	517 (61.7)	1679 (59.3)
Platinum, *n* (%)^b^	412 (49.2)	1387 (49.0)

Percent values will exceed 100 for the columns because of drug combinations used in a patient’s line of therapy

^a^Percent from total number of patients receiving first-line therapy

^b^Percent from total number of patients receiving second-line therapy

^c^Ns may be smaller as a result of consolidated regimen calculations

**Fig. 2:**
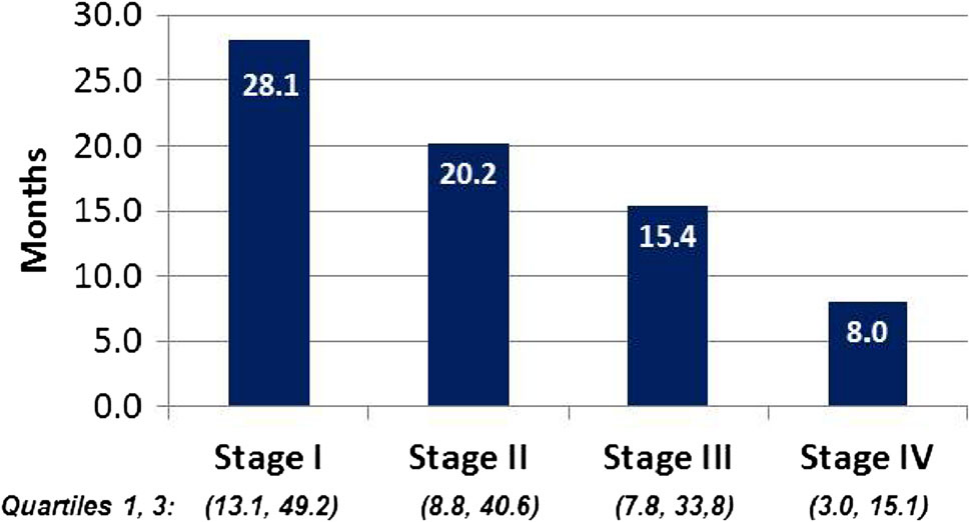
Median estimated survival by stage: electronic medical record data (*N* = 1533)

The duration of chemotherapy was also relatively brief. In the EMR data, first-line therapy was administered for an average of 63.2 days (SD = 64 days) and second-line therapy for an average of 57.3 days (SD = 75 days). In the administrative claims data, the duration of first-line therapy was an average of 53.5 days (SD = 63.4 days), and the duration of second-line therapy was 41.2 days (SD = 55.7 days). Given that most regimens are administered on an every 21- or 28-day cycle, this represents an average of less than three cycles of chemotherapy before the treatment was discontinued. The estimated survival of patients by stage at diagnosis is presented in Fig. 2. As would be expected, survival decreases with advancing disease stage. Estimated survival data were only available using the last record in the database as a proxy in the EMR data.

Resource utilization data were available in the administrative database only. Of those who received chemotherapy, surgical procedures were identified in 879 (16.6 %) patients during chemotherapy treatment and 1484 (28.0 %) outside the chemotherapy treatment period. Surgical procedures were identified in 328 (6.2 %) of those who did not have evidence of chemotherapy. Concomitant prescription medication use and hospitalization rates reflect the biology and burden of the disease and its treatment on patients. In the administrative data, 76.9 % (*n* = 4077) of patients treated with chemotherapy for gastric cancer received anti-emetics, 75.3 % (*n* = 3992) received pain medication, and 56.8 % (*n* = 3012) received antibiotics. During first-line therapy, 708 (13.4 %) patients were hospitalized. During second-line therapy, 8.6 % of patients (*n* = 247) were hospitalized. Emergency room visits were not uncommon during first- and second-line treatment. During first-line therapy, 991 (18.7 %) patients went to the emergency room. Many patients experienced multiple visits, with a total of 1787 visits recorded during first-line therapy. A similar pattern was evident in the second-line setting; a total of 345 patients (12.0 %) receiving second-line therapy experienced a total of 572 emergency room visits during the treatment period.

There was also considerable variability in the cost of the care of these patients, both from a third-party payer perspective and from a patient perspective (i.e., out of pocket costs). All-cause cost data during first- and second-line therapy are presented in Table 3. Patient out-of-pocket costs averaged $926.11 (SD = $1771.90) during first-line therapy and $646.93 (SD = $3262.49) during second-line therapy (Table 4). In addition to the costs incurred during chemotherapy, there were substantial costs of care following discontinuation of chemotherapy. An average of $80,148.07 (SD = $161,421.80) in all-cause total healthcare costs were incurred from the time of completion of chemotherapy to death or the end of the patient record in the database. For the 1630 patients with hospitalizations after completion of chemotherapy, the total cost of these inpatient stays was an average of $85,769.49 (SD = $178,096.47) per patient.

**Table 3:**
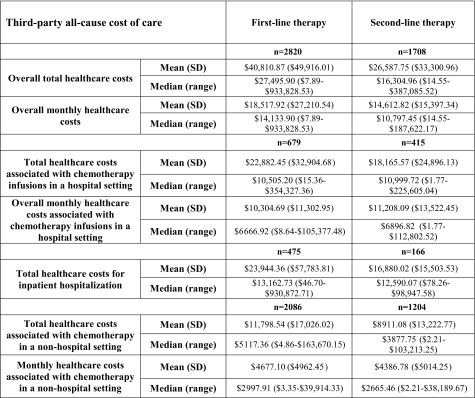
Third-party payer costs for gastric cancer patient care

**Table 4:**
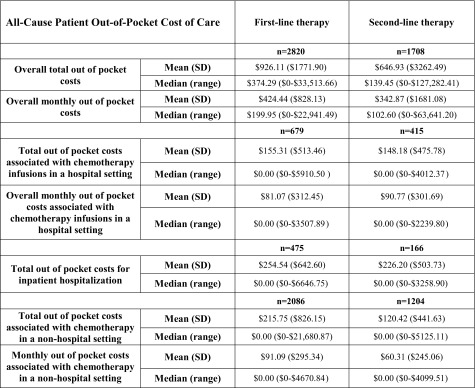
Patient out-of-pocket expenses

## Discussion

Gastric cancer is a disease with poor survival outcomes and is associated with a lack of standard treatment strategies, particularly following first-line therapy. Patients and the healthcare system incur financial burdens associated with this disease, although the range of expenses is considerable, with some patients incurring no costs and others experiencing what could be detrimental financial burdens. Although specific analyses were not conducted comparing hospital versus physician office chemotherapy infusion because of the bundling of costs in the hospital billing systems, this study suggests there may be differences to the patient that are not concordant with the direction of cost burden to the third-party payer. The median out-of-pocket cost for cancer care to a patient is numerically lower in the hospital setting, yet is higher for the third-party payer, and vice versa. Additional study is needed to understand the impact of trends in the delivery of cancer care on patient healthcare expenditures.

Data used for this study were collected as part of routine clinical or business practices and were not designed to measure or study follow-up or longitudinal care. Identification of disease was only possible using ICD-9 codes, which do not record pathology information about the disease. Although we excluded gastrointestinal stromal tumors (GIST), which are traceable with ICD-9 codes, there are other subtle pathologies not detectable by ICD-9 coding that may have added clarity to the heterogeneity of treatments seen in this study. Subtle differences in the number of regimens or rates of post-first-line therapy in the different databases could be caused by differences in the data source, rather than actual treatment pattern differences, but it is not possible to investigate this given the limitations of the data sources. If a patient changed the site of care to an oncologist whose EMR data are not uploaded to the IMS system, the care of that patient would no longer be captured, in contrast to administrative data, when one knows if a patient’s coverage has ended and if the claims are no longer being captured in that system. Similarly, only required fields are routinely entered, and missing data are a concern when using EMR systems that do not require practitioners to complete all data fields. Required fields in EMR and claims data are limited and typically do not capture over-the-counter medication use, response to therapy, or dates of disease progression. Assumptions were required to be made for the date of death and line of therapy as a consequence of database limitations. Other than data collection issues inherent to EMR and administrative data, the findings of types of drugs and treatment patterns appear highly consistent, demonstrating the heterogeneity of treatment patterns in subsequent lines of therapy.

Despite the differences in data sources and their respective limitations, this study does provide details about the treatment and follow-up care of patients diagnosed with gastric cancer in the U.S. across very large sample sizes. Gastric cancer is diagnosed in only about 22,220 patients per year, so access to longitudinal data from a cohort of more than 16,000 patients is valuable and contains important insights. A strong, consistent finding is the lack of standardization of treatment regimens after first-line therapy. Although the majority of patients received platinum and/or fluoropyrimidines in the first-line setting, more than 350 unique treatment regimens were identified in the second line in the administrative data. The number of regimens (*n* = 131) was also high in the EMR data, but may be streamlined as a result of the more limited data provided on specific chemotherapy treatments administered to patients (e.g., claims data provide evidence of all antineoplastic drugs administered and billed). However, even when grouping by therapeutic class, the variability remained high. The regimens used in this study suggest that the care that gastric patients receive after disease progression or recurrence is largely not evidence based, and treatment varies considerably. Few patients received treatments supported by randomized trial data, which included taxanes and irinotecan during the study period, in the setting of second-line gastric cancer. It may be in part the consequence of the lack of strong phase III data that a large amount of heterogeneity was observed during this time period.

The survival data was estimated and the analysis was limited to a subset of patients with stage data at baseline. Although these data are directionally consistent with the trend in SEER (Surveillance, Epidemiology, and End Results program) data, which show 5-year relative survival rates of 64.1 %, 28.8 %, and 4.2 % for localized, regional, and distant disease, respectively [4], they are limited to a small portion of the study population with stage data and are likely not representative of the gastric cancer population. It is unknown if other factors (such as receipt of surgery or chemotherapy treatment) made it more likely for this field to be populated in the EMR data.

Several recent randomized trials have been published demonstrating improved survival outcomes in the second-line setting for single-agent therapy [5–8]. These trials have primarily evaluated single-agent docetaxel, irinotecan, docetaxel, and ramucirumab. In addition to ramucirumab monotherapy [5], research has also recently demonstrated improved survival for the combination of ramucirumab plus paclitaxel versus single-agent paclitaxel in gastric cancer [9]. It will be of interest to examine how the emergence of these phase III trial data and the availability of new FDA-approved products, such as ramucirumab, may influence treatment patterns in the future. The data collected for the current study preceded the availability of these publications. Future research should evaluate the influence of this new evidence in the treatment patterns for the second-line treatment of gastric cancer and could build on this work by studying comparative effectiveness of these treatment regimens in the second line. Future research could build on this initial work as well to understand the cost-effectiveness of various regimens and sequences of care to inform treatment decision making for the care of these patients.
